# Multidisciplinary care planning in the primary care management of completed stroke: a systematic review

**DOI:** 10.1186/1471-2296-9-44

**Published:** 2008-08-05

**Authors:** Geoffrey K Mitchell, Robyn M Brown, Lars Erikssen, Jennifer J Tieman

**Affiliations:** 1 Discipline of General Practice, University of Queensland, Brisbane, Queensland, Australia; 2 University of Queensland Health Sciences Library, Brisbane, Queensland, Australia; 3 Department of Palliative and Supportive Services, Flinders University, Adelaide, South Australia, Australia

## Abstract

**Background:**

Chronic disease management requires input from multiple health professionals, both specialist and primary care providers. This study sought to assess the impact of co-ordinated multidisciplinary care in primary care, represented by the delivery of formal care planning by primary care teams or shared across primary-secondary teams, on outcomes in stroke, relative to usual care.

**Methods:**

A Systematic review of Medline, EMBASE, CINAHL (all 1990–2006), Cochrane Library (Issue 1 2006), and grey literature from web based searching of web sites listed in the CCOHA Health Technology Assessment List Analysis used narrative analysis of findings of randomised and non-randomised trials, and observational and qualitative studies of patients with completed stroke in the primary care setting where care planning was undertaken by 1) a multi-disciplinary primary care team or 2) through shared care by primary and secondary providers.

**Results:**

One thousand and forty-five citations were retrieved. Eighteen papers were included for analysis. Most care planning took part in the context of multidisciplinary team care based in hospitals with outreach to community patients. Mortality rates are not impacted by multidisciplinary care planning. Functional outcomes of the studies were inconsistent. It is uncertain whether the active engagement of GPs and other primary care professionals in the multidisciplinary care planning contributed to the outcomes in the studies showing a positive effect. There may be process benefits from multidisciplinary care planning that includes primary care professionals and GPs. Few studies actually described the tasks and roles GPs fulfilled and whether this matched what was presumed to be provided.

**Conclusion:**

While multidisciplinary care planning may not unequivocally improve the care of patients with completed stroke, there may be process benefits such as improved task allocation between providers. Further study on the impact of active GP involvement in multidisciplinary care planning is warranted.

## Background

Stroke covers several processes that cause permanent damage to a part of the brain by obstruction to cerebral blood flow or intracranial haemorrhage. This causes impaired physical function, including paralysis, loss or distortion of higher-order functions like language, memory and personality, and alteration of automatic functions like swallowing, speech, and continence. Significant stroke leads to major disability. Stroke is the third largest cause of death in Australia. In 1997, the annual cost of stroke care was $A555m, with the average cost of care twelve months after a first time stroke being $A18,956, and the lifetime cost being $A44,000 per case. Stroke care accounted for around 269,000 GP consultations per annum in Australia [1].

Coordinated care approaches, which aim to provide comprehensive care specific to the needs of the individual patient or patient group, are presumed to offer benefits to those with complex needs such as those recovering from stroke. Different approaches include case management, disease management programs or integrated service delivery [2-4]. Multidisciplinary care planning is one of these approaches.

Because of the range and seriousness of the impacts of stroke on patients and their families, multidisciplinary care is frequently required[5]. Such care enables participation by health professionals from different disciplines, services or sectors in planning and/or delivering care. However, given structural differences in the way hospitals and community agencies work, implementing multidisciplinary care across the primary/secondary divide can be exceedingly difficult to achieve.

The aim of this systematic review is to assess the impact of co-ordinated multi-disciplinary care planning involving primary care professionals, either wholly within primary care or by primary-secondary care teams, on outcomes in stroke, relative to usual care.

The approach to the investigation of the review was derived from the work of Mays, Pope and Popay [6] in describing frameworks for the review and synthesis of health services evidence which have to deal with different study designs, research traditions, theoretical orientations, and disciplines. Interventions of this nature are complex and context dependent, creating challenges in identifying and comparing studies. Studies are rarely replicated exactly. Analysis is question-driven, enabling examination of underlying context issues and background assumptions as well as the outcomes of a study.

This approach is not dissimilar in intent to other approaches to reviews of complex interventions such as Medical Research Council's Framework [7] where the emphasis is on the process relating to developing and evaluating a complex intervention or Theory of Change [8] which looks at the function and purpose of the intervention rather than the compositional elements alone. As it may be impossible to apply the method of one study to another context completely, these approaches enable systematic reviewing of interventions which may have multiple components, multiple outcomes and different implementation strategies.

## Methods

As health service interventions are multifaceted and are variously affected by the presence or absence of an evidence-base, methods used, stakeholders and contextual factors, to appreciate the impact of multidisciplinary care planning interventions better, we not only examined effectiveness by reviewing controlled trials, but also extended the scope to capture participants' views on how multidisciplinary care operates, and clinical experience (by reviewing qualitative studies, editorial opinion, and grey literature reports, case studies).

Evidence-based guidelines were examined to identify how multi-disciplinary care planning was best conducted, who should be involved and what roles participants should play.

Given the heterogeneity of study types, models of care planning and team organisation and outcome measures, a narrative approach to review and analysis of extracted data was undertaken.

### Search Strategy

We performed a computerised search of MEDLINE (from January 1990 to December 2006), EMBASE (from January 1990 to December 2006), CINAHL (from January 1990 to December 2006), the Cochrane Library (Issue 1 2006), and grey literature from web based searching of web sites listed in the CCOHA Health Technology Assessment list. The search combined synonyms for 'stroke' with 'primary care' and the following terms relating to methods/delivery of multidisciplinary care:

1. Managed care

2. Health care delivery

3. Patient care management

4. Patient care planning

5. Integrated care

6. Delivery of health care, Integrated (MeSH)

7. Critical pathways (MeSH)

8. Patient care planning (MeSH)

9. Patient care management (MeSH)

10. Patient care team (MeSH)

11. Multidisciplinary care team

12. Case management (MeSH)

The reference lists of identified articles were hand searched for relevant articles. [See Additional File 1]

### Inclusion criteria

Study types: RCTs, non-randomised trials, observational studies, qualitative studies

Intervention: Included any multidisciplinary planning process with GP as a participant or leader, mainly involving face to face or teleconferenced meetings, but not excluding other models.

Setting of care: Primary care

Patient population: Adults with a completed stroke

### Exclusion criteria

Studies of interventions targeted at health professionals only, such as education on stroke and stroke care for GPs were excluded.

Selection required that GPs played an active role in the discharge planning process. Thus studies where the GP participation comprised passive communication alone, such as a letter to the GP informing them of patient discharge, or where a multidisciplinary approach to discharge planning did not involve primary care professionals, were excluded.

We also included guidelines and articles featuring narratives/commentaries on multidisciplinary approaches to management in primary care. However, guidelines that described the tasks of general practice, but did not examine their role in multidisciplinary planning were not considered. Study outcomes were not used as selection criteria. Thus, a range of endpoints relating to processes of care, biomedical status and self-report measures are included.

### Data collection and analysis

Two independent reviewers applied the selection criteria, and extracted data. The reviewers each examined all abstracts from the initial search. This led to the retrieval of full-text articles, which were read by both researchers. A quality assessment was conducted, using the Cochrane Quality assessment method for controlled trials [9] and a quality assessment method devised by Aoun and colleagues [10] for qualitative studies. Final articles for inclusion and quality assessment were decided by consensus.

## Results

### Number of papers

We identified 1045 papers. After discarding duplicates and irrelevant articles, and reading the full text of the remaining papers, we included 18 papers. These included five papers reporting RCTs (three papers are based on one trial and a further paper where a variant of the program was delivered. This is considered a separate trial because the rural setting meant different types of interaction between primary and secondary professionals.), seven qualitative studies, including three that described care models and one describing a financial analysis, and six papers that included guidelines and local care models. There were no systematic reviews that covered the topic of interest. Almost all articles arose from secondary care sources.

Details of the included papers are found in Tables 1, 2, 3.

**Table 1:** Details of Randomised Controlled Studies of multidisciplinary care in the community care of stroke

**Author, year, location,**	**Study participants**	**Participants (intervention/control)**	**Test and control groups similar at baseline?**	**Patients lost to follow-up (intervention/con trol)**	**Patients without complete data (intervention/control)**	**Intervention**	**Study designs**
Askim et al 2004 [(20) Norway	Stroke patients being discharged to rural areas.	31/31	**Yes**			Home based care coordination from hospital specialist unit for four weeks post- discharge for rural areas. Primary health care providers visited home, phone liaison with stroke unit to develop care plan.	RCT
			Age, Diagnosis, Predisposing medical history	Died at 52 weeks 8/5	Withdrew		
			Functional status		<6 wks 0/1		
					<26 wks 0/2		
					<52 wks 0/2		
			**No**				
			nil				
Faberberg et al 2000 [(19) Sweden.	Acute Stoke patients	166/83	**Yes**			Stroke unit care including comprehensive discharge planning (includes "contact with primary care services" unspecified) vs general ward care	RCT
			Gender				
			Age	Died			
			Medical history except angina	3 wks 14/8			
				3 mths 7/5	3 wks 12/1		
			Living alone	12 mths 23/6	3 mths 4/4		
			Conscious state on admission	Alive at 12 mths 70/71%	12 mths 0/4		
			Nature of stroke				
			Final diagnosis				
			**No**				
			Angina 27/15% (P = 0.036)				
Trondheim study(21, 29, 30) Norway	Acute Stroke patients	160/160	**Yes**			Early supported discharge from specialist stroke unit. Includes comprehensive coordination with primary care services	RCT
			Age	Died			
			Gender	Initial admission 4/5			
			Living alone	6 wks 4/7			
			Medical history	26 wks 13/15			
			Functional state	52 wks 0/0			
			**No**				
			Nil				

**Table 2:** Details of Qualitative Studies of multidisciplinary care in the community care of stroke

**Study Authors**	**Study group**	**Numbers**	**Objective**	**Method**	**Outcomes**
Allen et al 2002[(14, 15) UK	Stroke at discharge to home	8	To describe the micro-management of the Discharge process	Ethnographic	Staff on ground prepared to bend rules to get the best outcome for patients. Very staff dependent. GPs are part of this process, but not to a wide extent.
Brotheridge et al 1998[(13) UK	Stroke patients and carers post discharge	30	To describe GP care experienced by stroke patients after discharge in the community	Separate interview of patients and carers	GP care reactive and often confined to treating complications and writing scripts. Sometimes had to ask to be seen, Patients and carers disappointed by GP response, expected more coordination.
Sackley and Pound, 2002[(24)	Health carers of stroke patients about to enter nursing home.	12	Describe process of setting discharge priorities for patients about to enter nursing home	Delphi process, plus two group meetings	Ranking of goals in physical care needs, care needs (ie process needs), he discharge process. Rankings changed through the process.

**Table 3:** Studies that describe the processes of care of multidisciplinary care in the community care of stroke

**Author, year, location**	**Purpose of paper**	**Study design**	**Outcomes**
Geddes et al, 2001[(16) UK	Describe six examples of models of care of home-based rehabilitation of stroke patients.	descriptive	A taxonomy of four models (See Figure 1.), describe outcomes across models for 1076 patients in 1998
Henderson et al, 2001[(22) UK	Describe cost differentials for stroke rehab conducted in secondary hospitals, and in semi-rural country hospitals under local team care	economic	Reduction of costs from UK 183000 pa to UK 74000 pa in this district.
McBride, 2004[(17) Canada	Describes care given by nursing care coordinator in the community post stroke.	Descriptive, model developing	Six main areas of care: negotiating health system (including liaison role, pt safety, behavioural, physiological: complex; Family issues; physiological: basic.

### Quality of papers

RCTs scored a mean of 7.8/10 using the Cochrane Quality scoring system [9]. The qualitative papers which were scored averaged 12.4/16 on the Aoun quality scoring system [10].

### Theoretical assumptions

It was difficult to establish the theoretical basis for most studies due to the brevity and general nature of introduction and methods sections. Several studies did note the intervention related to an existing care program or one that had been used in another setting or with another population [11-13]. No study outlined a specific theoretical framework that proposed mechanisms by which expected outcomes would be achieved. One qualitative study examined discharge planning ethnographically, drawing conclusions about the processes employed and what these processes relied on [14,15], while two were involved in describing or developing discharge planning models [16,17].

### Models of care

**Figure 1:**
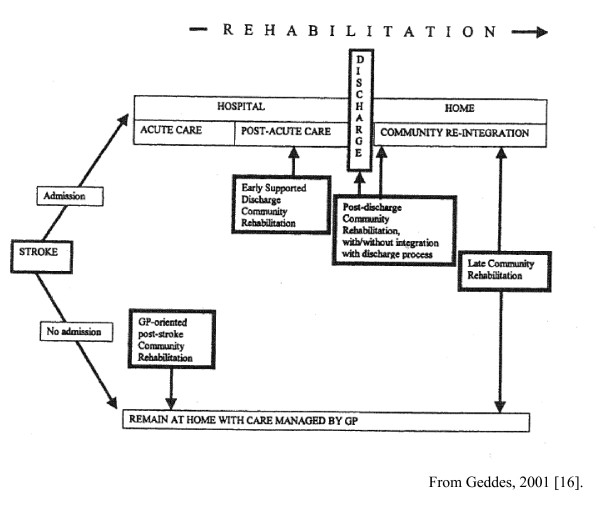
**Primary Care Models for management of community-based stroke patients [See additional File**2**].**

Four types of models of care coordination/care planning were described. These are represented in Figure 1 (copyright information available in additional file 2). The relative effectiveness of the different types of models on outcomes could not be assessed.

Secondary outreach was the dominant model reported. Here patients were followed after discharge by care coordinators (usually nurses) based in specialist units (eg [17,18]), and working in the community with allied health personnel based in the same unit, or with local primary care providers. Teams usually comprised of a specialist physician, the patient's general practitioner (GP), nurse, physiotherapist and occupational therapist, with others like speech therapists and administration staff being included in some teams. In some models, decisions are made and enacted by the specialist team [16,19]. In others, there is a deliberate decision to transfer care and responsibility to the primary care providers. This was done by supported discharge [20,21] by transfer of patients to a GP led, community hospital based team [22], or to provide services to patients with stroke who never left the care of the GP, and did not get admitted to hospital in the acute phase [16].

Communication between specialist and primary caregivers thus ranged in complexity, from a phone call to the GP and primary service providers after treatment decisions had been made [19] to structured case conferences where patients, carers, secondary care providers and primary care providers met multiple times to develop a care plan and apportion responsibility for the required tasks [20,21].

While system level policy sets parameters for patient management, how these are enacted is determined at "street level" [23]. Allen et al describe the process of discharge of stroke patients from hospital to home in Wales [14,15]. The process is defined by multiple service providers, complex funding arrangements and role definitions. Staff involved have many multi-professional meetings and case conferences on an apparently regular basis. Staff members draw on their experience of the system, knowing how to massage rules to meet the individual needs of their clients. This system relies on the goodwill, motivation and corporate knowledge of the staff concerned, and is thus placed at risk by staff turnover.

Finally, a variant of multidisciplinary care planning occurs where a multidisciplinary team develops generic guidelines for patients, and these guidelines are implemented as routine care in a community setting[24].

### The role of primary care

Primary care fulfils many roles within the health system including acting as a first contact point and a gateway to health care services. It is also increasingly taking on a role in coordinating ongoing and complex care needs. It has been noted that these roles can create different perceptions of primary care – one where it is largely autonomous and an independent entity within the health care system and the other where it is the front door or stepping stone to the broader suite of health care resources [25]. This can lead to an expressed or implicit set of professional tensions around respective roles of different providers.

Successful outcomes from multidisciplinary care planning assumes that each member of the team understands, accepts and actually carries out the tasks deemed necessary to maximise the patient's function and quality of life. However, while it may be assumed by secondary care staff that primary care routinely undertakes tasks that are outlined by clinical guidelines, this may not necessarily be what actually happens.

For example, guideline documents written by specialist doctors [11,12,26] ascribe many roles and tasks to general practitioners in post-stroke rehabilitation. These include the prevention and treatment of the complications of stroke, preventive health to minimise the risk of subsequent strokes, identifying the need for further rehabilitation when progress slows (so-called "refresher rehabilitation"), and acting as a trusted advisor for the patient in such sensitive areas as resumption of driving and sexual activity.

However, GPs may not be not filling these roles [13]. Stroke patients and their caregivers report that general practice care is reactive- responding to requests for prescriptions, or to an emergency situation or complication. This is not what is anticipated and needed by patients, researchers and policy makers with respect to chronic long term care conditions.

A study that developed a taxonomy for the tasks of a nurse coordinator in the six weeks post-discharge [17] found six major work themes: (dealing with the) Health system, Safety, Behavioural, Physiological (complex) (eg cognition, balance), Family, and Physiological (basic) (eg eating, elimination, movement). Coordination of services to achieve these aims was the main challenge of the job.

### Patient and Carer outcomes

The results of trials designed to test multidisciplinary care are presented in Table 4.

**Table 4:** RCT outcomes

**Study**	**Patient group**	**Outcome measure**	**Estimate of treatment effect (Intervention/Control)**	**Result (P values if ≤ 0.05, or reported 95% CIs^a^)**
**1. Function**				
Askim	Stroke patients discharged to rural areas	Independent at		
		6 wks	51.6/51.6%	NS^b^
		12 months	39.0/52.0%	NS
Fagerberg	Stroke patients requiring hospitalisation			
		Barthel Index		
		Baseline	44/42	NS
		3 wks	71/67	NS
		3 mo	80/79	NS
		12 mo	82/76	NS
		At home		
		3 wks	46/44"%	NS
		3 mo	68/61%	NS
		12 mo	61/59%	NS
Trondheim Study Ingeberg	Early discharge from stroke unit vs normal care	% at home		
		post initial admission	64.4/45.6%	P 0.001
		6 wks	74.4/55.6%	P 0.0004
		26 wks	78.8/73.2%	P 0.239
		52 wks	75/68.8%	P 0.265
		Independent at		
		26 wks	65/51.9%	P 0.017
			Rankin Scale <2	OR^c ^1.72 (1.10–2.70)
			Barthel Index <95	1.54 (0.99–2.39)\
		52 wks	Rankin Scale <2	OR 1.56 (1.02–2.44)
			56.4/44%	
2. **Mortality**				
Fagerberg	Stroke patients requiring hospitalsation	3 wks	9/10%	NS
		3 mo	13/16%	NS
		12 mo	27/23%	NS
Trondheim	Early discharge from stroke unit vs normal care	6 wks	2.5/4/4%	NS
		26 wks	8.1/9.4%	NS
		52 wks	13.1/16.3	NS
**3. Quality of Life**				
Askim	Stroke patients discharged to rural areas	Nottingham Health Profile		
		Seven subscales		
		6 weeks		NS
		26 weeks	Social isolation subscale	0.046
			Others (6 subscales)	NS
		52 weeks		NS
		Caregiver Strain 'Index		
		6 weeks		NS
		26 weeks		NS
		52 weeks		NS
Trondheim	Early discharge from stroke unit vs normal care	Global Nottingham Profile		
		52 wks		
		Part 1	81.6/78.9 (median)	0.048
		Part 2	84.3/79.4	0.073
		Caregiver Strain index		
		52 wks	Trend favours intervention	0.076
**3. Service utilisation**				
Trondheim	Early discharge from stroke unit vs normal care	Days as inpatient 52 wks	18.6/31.1	0.032

^a ^CI Confidence Interval

^b ^NS not significant

^c ^OR Odds Ratio

The study trials were heterogenous in structure and outcomes and therefore were not suitable for meta-analysis. The results are presented in narrative form. Four outcome measures were described in the various studies – function, mortality, quality of life and service utilisation.

#### 1. Function

There were mixed results for the proportion of stroke patients being discharged home. There were improvements in the Trondheim study intervention (which featured early discharge and intensive care planning involving primary care teams including GPs) but no differences in other studies. One tested stroke unit care including intensive discharge planning, which included contact with primary care providers (nature unspecified). The other study of rural patients involved an assessment by locally based primary caregivers of the patient's home environment liaising with the tertiary hospital's specialist team by phone. There were improvements in the proportion of people achieving independence at 26 and 52 weeks in the Trondheim study, but none seen in the other two studies. Trondheim patients were home in greater proportions and at an earlier stage than controls.

#### 2. Mortality

There was no difference between intervention and control group mortality in any of the studies.

#### 3. Quality of Life

Interventions that include multidisciplinary care planning may have improved quality of life. The Trondheim studies showed improvements in quality of life indices at 12 months. The Askim study had improvement in the social subscale of quality of life (the only one of seven indices to show statistically significant changes) at 26 weeks, and described this as a trend towards improved quality of life. Neither study showed reductions in caregiver strain, though there was a trend favouring intervention in the Trondheim study at 52 weeks.

#### 2. Service utilisation

There were reduced bed day numbers in the twelve months post-discharge in the Trondheim study.

A financial analysis of rehabilitation of post-stroke patients in semi-rural community-based (GP-run) hospitals in Scotland compared with secondary care found a reduction in cost of 60% [22].

## Discussion

The use of a systematic review to describe the outcomes of a complex intervention has to take into account the context in which the study was performed [6,27]. This review has identified various models of care planning and coordination that have been utilised in stroke management in the primary care setting. National health systems use an array funding and administrative structures which support models of care provision. However, two basic coordination approaches emerge. The first assumes that clinical decision making is a specialist task, and the primary care role is one of facilitating the implementation of the care plan and timely identification of complications. The second overarching model assumes transfer of care and responsibility to the primary care team, and use techniques that engage actively with primary care providers with transfer of care in mind. Prevention of secondary stroke as a primary care responsibility is common to both models identified and is acknowledged in stroke guidelines [28].

It is uncertain whether multidisciplinary care involving GPs improves outcomes in patients with completed stroke. Interpretation of the results is difficult, as results of the two largest studies appear contradictory, and analysis is complicated by the diversity of outcome measures. The Trondheim study tested the hypotheses that close cooperation between specialist team and primary care team was beneficial for the patient's recovery, and that rehabilitation conducted at home was more beneficial than rehabilitation unit based care. This was the study that improved the proportion of patients achieving independence (with a number needed to treat of seven) and modest improvement in quality of life at twelve months [21,29,30]. By contrast, the other large study aimed to compare the effectiveness of conventional care with specialist stroke unit care including intensive discharge planning: this trial had limited involvement of primary care professionals [19]. It showed no differences between intervention and control groups. The smallest study applied to rural patients [20]: there were also limitations to the degree of integration of specialist and primary care services. It too showed no differences in outcomes.

Secondary care providers may make assumptions about what should be happening at a primary care level without systems to check the presumed care actually happens. Particularly when there is patient follow-up in the community by a secondary service, primary care service providers may not know what their role is expected to be, without dialogue and negotiation.

The question thus arises whether systematic effort put into engaging GPs and other primary care providers is effort well spent. While it is difficult to draw conclusions, there may be at least process benefits. It makes sense to ensure that primary and secondary care providers know what their respective responsibilities for the care of the patient are. Without this, some essential tasks assumed to have been done by another professional may be missed, while other tasks are duplicated.

Education is one avenue to increase GP's knowledge of stroke care requirements, but outcomes rely to some extent on the GP's own areas of interest [31] and mode of delivery [32,33]. Case-based education relating to problems experienced by current patients is effective in improving knowledge and practice. Engaging primary care providers including GPs in multidisciplinary discharge planning thus provides excellent opportunities to use the index case to teach.

Implementation of change in care approaches seems to depend on professionals working collaboratively. Finding mechanisms and forms of collaboration that support patient outcomes is an increasing important area of implementation research. The Alberta Primary health care project report identified eight critical factors for forming successful teams including "involving all members in planning and coordination" and "defining roles and responsibilities" [34] Recent work suggests that personal relationships between specialists and primary care providers are essential for improved communication [35].

There are limitations to this study. Frameworks for the review and evaluation of health services and care programs are still developing. The interventions and outcomes of the included studies in this study are heterogeneous, and therefore the review presents the findings in narrative form only. Although a comprehensive search strategy was undertaken, given the complexity of possible sources, terminology and brevity of descriptions of interventions, some literature may have been missed. As there is no single definition of the tasks and composition of multidisciplinary care planning, inappropriate inclusion or exclusion of a study may have occurred. Dual review for inclusion sought to address this issue. Although all included studies had multidisciplinary care planning as part of the interventions assessed, different processes and arrangements meant that it was not possible to determine the degree to which multidisciplinary care planning as an independent element led to the observed outcomes.

## Conclusion

It is unclear whether coordinated care planning involving GPs and primary care health professionals makes an unequivocal difference to patients outcomes to patients with completed stroke. The inconsistency in results suggests that further study around the type of involvement of GPs in the multidisciplinary planning process could be illuminating.

## Abbreviations

MeSH: Medical Search Headings; GP: General Practitioner; Wks: weeks; Mths: months; pa: per annum; CI: Confidence Interval; NS: Not significant; OR: Odds Ratio.

## Competing interests

The authors declare that they have no competing interests.

## Authors' contributions

GM conceived this systematic review, devised the search strategy, provided the initial analysis of the data and coordinated writing of the article. RB conducted the literature searches, and with GM assessed which articles should be included. LE searched the grey literature and provided advice on the search strategy. JT managed the project of which this review was a part, provided conceptual advice on the conduct of the project, and provided extensive editorial input into the final manuscript. All authors provided input into the interpretation of the data, and provided editorial input into the final manuscript.

## Pre-publication history

The pre-publication history for this paper can be accessed here:



## Supplementary Material

Additional file 1 Supplementary statement re evidence. Provides additional information on the process used to extract data from literature searches. Click here for file

Additional file 2 Sage Permission Form. Copyright permission for the figure presented as Figure 1. Click here for file
