# Supplementary material for: Multidisciplinary care planning in the primary care management of completed stroke: a systematic review
Source: BMC Fam Pract. 2008 Aug 5;9:44. doi: 10.1186/1471-2296-9-44 (PMC2518150; doi:10.1186/1471-2296-9-44)
Supplement: Additional file 1 — Supplementary statement re evidence. Provides additional information on the process used to extract data from literature searches. [file 1471-2296-9-44-S1.doc]

The following assessments were used for qualitative and quantitative studies.

Higgins JPT and Green S. Cochrane Handbook for Systematic Reviews of interventions (Version 4.2.5, updated May 2005).

In The Cochrane Library, Issue 3, 2005. Chichester, UK: John Wiley and Sons Ltd.

The scoring we used to judge the quality of randomised trials was derived from Chapter 6 of the Cochrane Handbook of Systematic Reviews. This chapter describes four sources of potential bias: Selection, Performance, Attrition and detection. It also describes a simple summary description of the Degree to which a study design minimises the risk of these biases. (Table 1) We have allocated a score of 2(low risk or bias), 1 (Moderate risk of bias) or 0 (high risk of bias) for each source of bias, after carefully considering the features that indicate possible bias.


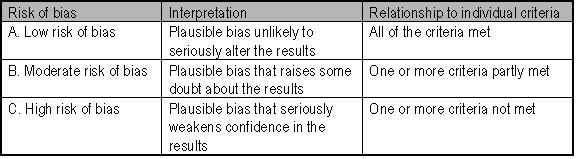


The questions we asked were:

Selection bias: Were the test and control populations similar?

Was there contamination of intervention by control subjects?

Performance Bias: Was the allocation of the subjects concealed to them and the clinicians?

Attrition Bias: Were ≥80% of subjects in each group followed?

Detection Bias: Was assessment blinded to allocation?

**Rating system for qualitative evidence**

(Aoun MJA 183: 264-266)

Studies were scored for Quality of evidence (quality of evidence used to minimise bias): This was assessed with eight questions, each with a yes/no answer (scored as 1 or 0 respectively). Then two questions relating to the strength of the evidence and the relevance of the study to the question of interest ( 4 points each). Hence the maximum possible score is 16.

Quality questions:

- Was the aim of the study clear?
- Was the paradigm (philosophical and scientific approach, such as logical positivistic, qualitative) appropriate to the aim?
- Wan the methodology (overall qualitative approach, such as phenomenological, grounded theory, critical theory) appropriate to the paradigm?
- Were the methods (e.g. sampling, data collection, analysis) appropriate to the methodology?
- Could the rigour of the study be established? (i.e. were the methods explicit and transparent, did researchers make explicit their own beliefs, did the analysis search for “negative” cases?)
- Did the sampling strategy address the aim?
- Was the data analysis appropriately rigorous?
- Were the findings clearly stated and relevant to the aim?

Strength of the evidence (magnitude of the intervention effect):

4-very high; 3- high; 2- low; and 1- very low

Relevance of the outcome measures and the applicability of the study results to the clinical question:

4- very relevant; 3- relevant; 2- of some relevance; 1- of little or no relevance
