# Supplementary material for: Multidisciplinary care planning in the primary care management of completed stroke: a systematic review
Source: BMC Fam Pract. 2008 Aug 5;9:44. doi: 10.1186/1471-2296-9-44 (PMC2518150; doi:10.1186/1471-2296-9-44)
Supplement: Additional file 2 — Sage Permission Form. Copyright permission for the figure presented as Figure 1. [file 1471-2296-9-44-S2.doc]

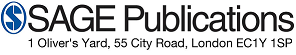


**<Your Address>**

Dear Sir or Madam,

Thank you for your permission request as set out below.

**__________________________________________________________________________________________**

**DETAILS OF Sage Publications material Required:**

**•Material: Figure 1 from:** Geddes JM, Chamberlain MA. Home-based rehabilitation for people with stroke: a comparative study of six community services providing co-ordinated, multidisciplinary treatment. Clinical Rehabilitation. 2001 Dec;15(6):589-99.

**__________________________________________________________________________________________**

**DETAILS OF YOUR PUBLICATION:**

**• Publication Type:** journal article

**• Publication Title:** Mitchell G, Brown R, Eriksson L, Tieman J. Multidisciplinary care planning in the primary care management of completed stroke: a systematic review**.**

**• Publisher:** BioMed Central (BMC General Practice)

**• Publication Date:** Journal article being submitted

**• Your reference:** N/A

**• Rights Required:** electronic internet

**• Language:** English

**• Course title & duration:** N/A

**• Number of copies to be made:** <fN/A

**• Vat number (EU only): N/A**

**__________________________________________________________________________________________**

**TO BE FILLED OUT BY SAGE PUBLICATIONS:**

**• Our reference:**

**• Fee:**

**• Acknowledgement required:**

**__________________________________________________________________________________________**

Sage Publications is pleased to grant a **non-exclusive license** for this use. Permission is granted only as set out above, subject to payment and subject to proper acknowledgement. Separate permission should be sought for any further copies, use or edition. Permission does not include any copyrighted material from other sources that may be incorporated in the selection.

**Please inform the authors of this re-use. Use of the material constitutes acceptance of the terms on this and the accompanying page**

Sage will issue you with a formal invoice for payment which must be settled within one month of receipt.

Thank you for your interest in Sage Publications.

Yours sincerely,

Rights & Permissions Department

Sage Publications Ltd (UK)

[books-permissions@sagepub.co.uk](mailto:books-permissions@sagepub.co.uk)

[journals-permissions@sagepub.co.uk](mailto:journals-permissions@sagepub.co.uk)

[www.sagepub.co.uk](http://www.sagepub.co.uk/)


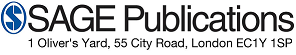


**Terms of Agreement:**

**Sage Publications will assume this material will be used once permission has been issued unless we are specifically informed otherwise.**

**Print Permissions:**

Based on the information provided by you, non-exclusive world rights in the English language only for the afore mentioned rights are granted subject to the following conditions:

No deletions from, additions to, or changes in the text may be made without the written consent of Sage Publications and the author. Permission does not include any copyrighted material from other sources that may be incorporated in the selection.

**Electronic Permissions: (this includes any permission with an electronic aspect)**

Based on the information provided by you, non-exclusive world rights in the English language only for the afore mentioned rights are granted subject to the following conditions:

No deletions from, additions to, or changes in the text may be made without the written consent of Sage Publications and the author. Permission does not include any copyrighted material from other sources that may be incorporated in the selection.

If this permission is not renewed, the material must be removed from your electronic reserve at the end of the permission period.

Access will be restricted to authorised users who require this material via your electronic reserve. Please ensure that the Sage Publications web site homepage address: [www.sagepub.co.uk](http://www.sagepub.co.uk/) is included with the electronic file. You also assert that you have sufficient control over your electronic reserve to prevent further use of the material without proper acknowledgement or further permission from copyright holder.

Please include the following statement on your electronic reserve:

‘Copyright Agreement: All material included in the PDF samples below is the exclusive property of the Sage Publications, or its licensors, and is protected by copyright and other intellectual property laws. The download of the file(s) is intended for the User’s personal and non commercial use. Any other use of the download of the Work is strictly prohibited. User may not modify, publish, transmit, participate in the transfer or sale of, reproduce, create derivative works (including course packs) from, distribute, perform, display, or in any way exploit any of the content of the file(s) in whole or in part. Permission may be sought for further use from Sage Publications Ltd, Rights & Permissions Department, 1, Oliver's Yard, 55, City Road, London EC1Y 1SP, UK Fax: +44 (020) 7324 8600. By downloading the file(s), the User acknowledges and agrees to these terms.’

**Photocopying Permissions:**

Based on the information provided by you, non-exclusive world rights in the English language only for the afore mentioned rights are granted subject to the following conditions:

No deletions from, additions to, or changes in the text may be made without the written consent of Sage Publications and the author. Permission does not include any copyrighted material from other sources that may be incorporated in the selection.
